# Exploring healthcare professionals' attitudes to screening for disordered eating in type 1 diabetes

**DOI:** 10.1111/dme.70003

**Published:** 2025-02-13

**Authors:** Katie Fitzgerald, Christina Jones, Helen Partridge, Lindsey Rouse, Rose‐Marie Satherley

**Affiliations:** ^1^ Department of Psychological Interventions, Faculty of Health and Medical Sciences, School of Psychology University of Surrey Guildford UK; ^2^ Royal Bournemouth and Christchurch Hospitals NHS Foundation Trust – Bournemouth Diabetes and Endocrine Centre Bournemouth UK

**Keywords:** disordered eating, good health and well‐being, qualitative, type 1 diabetes

## Abstract

**Aims:**

Eating disorders and disordered eating behaviours are prevalent among adults with type 1 diabetes, making early identification essential for improving health outcomes. Although screening tools exist to detect disordered eating in type 1 diabetes (T1DE), their application in clinical practice remains limited. This study investigates healthcare professionals' perspectives and attitudes towards screening for disordered eating in adult diabetes services.

**Methodology:**

This qualitative study employed semi‐structured interviews with 13 healthcare professionals from adult diabetes services. Purposive sampling was used to ensure a broad range of healthcare professional viewpoints. The interviews centred on their perceptions of screening for disordered eating in type 1 diabetes.

**Results:**

Reflexive thematic analysis was used to identify four themes: (1) Uncertainty and Inevitabiltiy of T1DE: ‘A bit of a black hole’, (2) Asking about T1DE: ‘My fear is…am I overstepping the line’, (3) Patient‐provider relationships: ‘A backward step’, and (4) Lack of support for T1DE: ‘Where do you go?’. While healthcare professionals recognised the link between diabetes management and disordered eating, they lacked confidence in screening for these challenges due to limited time and the scarcity of specialist disordered eating resources for type 1 diabetes.

**Conclusion:**

This study highlights the challenges healthcare professionals face in screening for disordered eating in type 1 diabetes. By highlighting the need for enhanced training and clear clinical guidelines, this research suggests pathways to improve healthcare professional confidence in addressing these critical conversations with patients, ultimately supporting better health outcomes.

**What's new?**

- Eating disorders and disordered eating behaviours are prevalent among adults with type 1 diabetes (T1D), yet screening tools are underutilised in clinical practice.
- This study identifies healthcare professionals' reveal a lack of confidence in initiating discussions about T1D‐related disordered eating (T1DE), often relying on observable indicators (e.g. gender and weight) due to fears of patients' reactions and perceived lack of support for T1DE.
- Enhancing training on T1DE‐specific screening tools and effective communication, alongside establishing clear clinical guidelines and referral pathways, is essential for improving the identification of at‐risk individuals.

## 1 INTRODUCTION

Eating disorders are serious mental health conditions with specific diagnostic criteria^1^ and adolescents with type 1 diabetes (T1D) are more than twice as likely to develop an eating disorder compared to their peers.^2^ They are also 1.2 times more likely to engage in disordered eating behaviours that fall short of clinical diagnosis.^2^ This elevated risk has been linked to the dietary focus needed for T1D management.^3^ While no formal criteria exist for disordered eating in T1D, in the United Kingdom (UK), guidelines for identifying T1D‐related disordered eating (T1DE) have been developed.^4^ These include fear of weight gain, inappropriate insulin restriction, and compensatory behaviours that negatively impact health, cause diabetes distress, or impair daily functioning.

Identifying T1DE is critical, as it is linked with poor T1D outcomes and a threefold higher mortality risk compared to T1D alone.^5^, ^6^ Early detection and support improves outcomes,^7^, ^8^ but healthcare teams often lack confidence in identifying T1DE.^9^ Although several T1DE screening tools exist^10^ implementation remains slow^11^, ^12^ with documented barriers like healthcare provider (HCP) confidence and training in paediatric settings.^13^ However, little is known about T1DE screening in adult settings.

The long‐term nature of T1DE can lead to cumulative health complications, requiring ongoing management in adulthood.^14^ Adults with T1DE face distinct challenges, such as increased autonomy over diabetes care and unique psychosocial stressors, which may exacerbate disordered eating behaviours. This study addresses the gap by exploring HCPs’ perspectives and attitudes toward T1DE screening in adult T1D services.

## 2 METHODS

A qualitative design with a critical‐realist stance was adopted,^15^ which provided an opportunity to understand HCPs' realities informed by clinical guidelines and the health system, whilst providing the opportunity to capture personal beliefs and clinical experiences. This design not only enables exploration of HCPs' perspectives but also how these are enacted in their professional behaviours, providing actionable insights for improving screening practices.

### 2.1 Participants and recruitment

HCPs working in the UK National Health Service (NHS) with at least 6 months of direct contact with adults with T1D were eligible to participate. Those working in paediatric settings were excluded. Participants were recruited through adverts on social media (e.g. Facebook, Twitter), healthcare forums, and professional mailing lists. Purposive sampling ensured diversity across HCP roles. Data collection occurred between October 2022 and July 2023.

Critical realism emphasises understanding the underlying mechanisms and structures shaping human behaviour, emphasising the depth and richness of data rather than the number of participants.^15^ By conducting interviews with 13 healthcare professionals, we obtained rich, varied, and detailed narratives providing insights into their beliefs and experiences regarding T1DE screening. Each interview provided substantial information regarding their experiences, perspectives, and expertise, contributing to high information power,^16^ and offering a comprehensive understanding.

### 2.2 Procedure and data collection

Participants contacted the researcher (KF) via email, received detailed study information, and had the opportunity to ask questions before providing consent. Participants then submitted demographic information (age, gender identity, ethnicity, job title, and whether they had a diagnosis of diabetes) and scheduled a time for their interview. Interviews were completed on Microsoft Teams and were audio recorded, lasting between 45 and 60 min. All participants had video cameras on during the interview.

A semi‐structured interview guide (Data S1) was created with input from two non‐participating T1D healthcare professionals and pilot‐tested with three participants, whose data were included in the analysis as no changes were needed. The 60‐min interview covered clinicians' understanding of mental health and T1D, experiences with T1DE, use of screening tools in clinical practice, and views on discussing T1DE with patients. All participants received a £10 Amazon voucher for their participation.

### 2.3 Analysis

Audio recordings were transcribed, capturing communication nuances such as pauses or laughter. Identifiable information, such as names or locations, were redacted to protect anonymity. Transcripts were uploaded to NVivo to manage data analysis.

Reflexive thematic analysis^17^ began with data familiarisation, involving re‐reading transcripts and re‐watching video recordings to capture verbal and non‐verbal cues. This allowed for a deeper understanding of the context in which the data were generated. Transcripts were then open‐coded, considering both semantic meaning, and latent, underlying meaning, interpreted by the research team. Theme development involved discussion within the research team to identify, interpret and refine underlying patterns within the data. This process included iterative reviews of initial themes, where codes were examined and regrouped, focusing on the relationships between codes and ensuring themes provided the most accurate and meaningful interpretations of the data.

### 2.4 Researcher reflexivity

Reflexive thematic analysis highlights the researcher's role in active knowledge production.^18^ In this study, all researchers are female, with professional backgrounds in T1D healthcare and/or psychology. One has lived experience of T1D (RS) and another (KF) has personal experience of a bereavement due to diabetes. Interviews were conducted by a trainee clinical psychologist (KF) who had no prior relationship with the participants. KF's psychologist role may have influenced the way participants discussed mental health topics. Despite this, participants displayed a notable level of comfort and openness, suggesting the interviewer's professional background helped foster trust and facilitated rich data collection. The team engaged in reflexive practices to critically examine how their experiences and positions influenced their analysis, ensuring both individual and broader contextual factors were considered.

### 2.5 Ethics

The study was approved by the University of Surrey, Faculty of Health and Medical Sciences ethics committee (FHMS‐21‐22‐259‐EGA).

## 3 RESULTS

### 3.1 Participants

Thirteen HCPs (aged 28–60 years) completed interviews, with variable lengths of time working in T1D services (2.5–22 years). Participants held roles within specialised diabetes services, practising in multidisciplinary diabetes care teams. HCPs included 2 doctors, 4 nurses, 4 dietitians, 1 midwife and 2 psychologists. See Table 1 for details. Their professional responsibilities provided a range of perspectives on T1DE, including medical management, dietary behaviours and nutritional strategies, patient education, and close monitoring. The midwife, who specialised in T1D, provided insights specific to T1DE during pregnancy. The psychologists, specialized in mental health care for T1D and contributed expertise in mental health support tailored to this population.

**TABLE 1:** Participant characteristics.

Participant number	Job role	Age	Gender	Ethnicity
1	Consultant Diabetologist	45	Female	White British
2	Diabetes Specialist and Research Nurse	40	Female	White British
3	Diabetes and Endocrinology Registrar	31	Male	White British
4	Specialist Diabetes Dietitian	28	Female	White British
5	Diabetes Dietitian	45	Female	White British
6	Diabetes Specialist Nurse	60	Female	White British
7	Diabetes Specialist Dietitian	53	Female	White British
8	Diabetes Specialist Nurse	Not disclosed	Female	White British
9	Diabetes Specialist Dietitian	36	Female	White British
10	Senior Clinical Psychologist	38	Female	White British
11	Diabetes Specialist Nurse	54	Female	White Other
12	Diabetes Specialist Midwife	58	Female	White British
13	Senior Health Psychologist	32	Female	White British

### 3.2 Qualitative findings

Four themes were identified: (1) Uncertainty and Inevitability of T1DE: ‘A bit of a black hole’, (2) *Asking about T1DE*: ‘My fear is…am I overstepping the line’, (3) *Patient‐provider relationships*: ‘A backward step’ and (4) *Lack of support for T1DE*: ‘Where do you go?’

#### 3.2.1 Theme 1: Uncertainty and inevitability of T1DE: ‘A bit of a black hole’

HCPs described T1DE as a complex and widespread problem affecting the T1D community. They likened their struggle to understand T1DE to peer into ‘a black hole’, a metaphor aptly capturing the uncertainty and confusion surrounding T1DE. The complexity and subtlety of T1DE often led to oversimplified explanations and a sense of helplessness among HCPs, making T1DE feel uncontrollable. Participants conveyed a strong sense of inevitability regarding T1DE, reflecting a belief it is an almost unavoidable consequence of living with T1D. For example, one nurse noted how individuals with T1D perceive food through the lens of T1D management: ‘it's there in the forefront of [people with T1D's] head. It's not just a plate of food, it is a plate of carbohydrates, fat and insulin. I wonder if everyone with T1D will not have, therefore, some sort of being on that spectrum (P11)’. HCPs understand T1DE as an often inevitable part of T1D management, in combination with external factors out of their control, like sexual abuse and childhood trauma. P10 highlighted the ‘complex link between eating and diabetes and trauma’ and P2 suggested those with eating disorders have ‘gone through some traumatic experiences’. One consultant shared how this belief creates a reluctance among HCPs to engage with T1DE, adding to its perceived complexity: ‘underlying all of this is childhood sexual abuse, and […HCPs] don't want to go anywhere near that’ (P1). By framing T1DE as inevitable and primarily driven by external, uncontrollable forces, T1DE becomes detached from everyday clinical responsibilities. This externalisation renders T1DE more abstract and less actionable, reinforcing perceptions it cannot be managed within the scope of routine care.

Many participants highlighted reluctance to screen for T1DE as ‘we don't have enough knowledge’ (P11) and healthcare professionals ‘aren't confident because they haven't got that awareness or knowledge’ (P2). In their attempts to understand and identify T1DE, HCPs often focused on visible symptoms to make T1DE more tangible. Many defaulted to associating T1DE with young females, despite acknowledging the need to consider a broader demographic. One nurse reflected on this bias: ‘got to think about men as well as women. You tend to have that unconscious bias. You think, female springs to mind’ (P6). Furthermore, physical health indicators were used to identify T1DE, reflecting a focus on visible symptoms. One nurse mentioned immediately suspecting an eating disorder based on weight change: ‘[the patient was] quite, quite, quite a big…quite…large. I met him the other day and, my, the first thing I thought is he probably has an eating disorder because, the, the change (P11)’. This focus on visible changes reflects attempts to simplify the complex nature of T1DE, however, by concentrating on what is seen, HCPs may overlook less apparent manifestations of T1DE.

#### 3.2.2 Theme 2: Asking about T1DE: ‘Am I overstepping the line?’

Despite recognising the importance of addressing T1DE, many felt uncertain about how to navigate these sensitive conversations. A fear of ‘overstepping the line’ was rooted in concerns about making patients uncomfortable and potential negative repercussions. This hesitancy appeared compounded by awareness of the secrecy and shame often associated with T1DE, which HCPs perceived as a barrier to open dialogue.

HCPs tended to assume patients would not spontaneously disclose T1DE, placing the onus on them to initiate the conversation, which most found challenging: ‘I find it difficult to bring those kind of questions up’ (P9, Dietitian). One nurse highlights the paralysing effect of this uncertainty: ‘[professionals] don't wanna ask the questions because they know what the answers might be. But I think it leaves the rest of us terrified that that's not something we're able to address’ (P1). There was a prominent fear of negative patient reactions, whether anger or discomfort, perhaps due to participants’ perception of an association between T1DE and feelings of shame, which further compounded HCPs’ hesitation. Furthermore, all HCPs believed there was a ‘correct’ way to ask about T1DE but felt unable to achieve this.

Some HCPs felt screening tools provided safety, as they have ‘better questions, better ways of asking them’ (P3, Registrar). However, many also anticipated negative responses, fearing patients may become ‘cross’ (P1), questions ‘may be upsetting’ (11), or they may ‘roll their eyes’ (P4, Dietitian). One nurse expressed concern these tools may cause harm: ‘I feel like it's really provoking someone’ [P6]. Concerns were not based on direct experiences but rather on assumptions about patients' negative reactions at being ‘confronted’ (P7, Dietitian) with questions about something they are assumed to feel shameful of. This reliance on assumptions highlights a significant barrier to addressing T1DE—HCPs may project their own discomfort and fear onto patients, potentially preventing meaningful dialogue.

Given these fears, HCPs preferred ‘subtlety’ (P3) when discussing T1DE, opting for indirect questions that would not explicitly reveal their concerns. Preferred approaches involved framing inquiries around general well‐being rather than eating disorders: ‘this is really about you and how you're feeling. And, and, and not screening you for an eating disorder’ (P1). However, HCPs’ discomfort around identifying T1DE was not expressed with other sensitive topics such as weight or alcohol intake. This contrast suggests secrecy and shame tied to T1DE hold a power, perhaps linked to the uncertainty and complexity discussed in Theme 1.

When suspecting T1DE, HCPs often relied on external evidence to validate their concerns before initiating a discussion with patients. This approach was seen as a means to bolster HCP confidence and provide a rationale for addressing the issue. One participant described the process of gathering evidence as being like a detective: ‘Checking back with the pharmacy…So you've got some evidence to support your suspicions’ (P7, Dietitian). Another described this process as ‘a solid piece of evidence almost to back up why you're asking that question and then kind of makes you feel more confident’ (P11, Nurse). While this approach could provide a sense of security, it also suggests that HCPs may need more confidence and support in trusting their clinical judgment.

This theme highlights the significant fears HCPs face when discussing T1DE. The apprehension around asking about disordered eating stems from concerns about discomfort, negative reactions, and potential harm, and is further intensified by associations between T1DE and secrecy and shame. These fears, often based on assumptions rather than experience, contribute to a tendency to avoid or approach the topic with extreme caution. This highlights a need for clear guidance and support to help HCPs confidently navigate these conversations.

#### 3.2.3 Theme 3: Patient‐provider relationships: ‘A backward step’

HCPs’ accounts emphasised the role of the therapeutic relationship in shaping their approach towards T1DE screening. For example, P13 (Psychologist) highlighted the importance of trust and rapport in creating a safe space for patients: ‘I try to foster that atmosphere or that safeness to be quite open’. This sentiment was echoed by other professionals: P11 (Nurse) mentioned needing ‘more rapport’ with patients before T1DE screening, while P1 (Doctor) stated, when a strong relationship is established, ‘patients are usually quite honest’ about their eating behaviours. This contrasts with the prior theme emphasising subtlety, suggesting a strong therapeutic relationship can make direct conversations more feasible and effective and supports individuals with T1DE in discussing behaviours they may feel shameful of. However, pressure to ensure patients’ physical safety and the constraints of limited time in clinical settings appeared to compromise the quality of relationships. Without the foundation of trust, abrupt questions about T1DE felt like a ‘backwards step’ threatening the patient‐provider relationship.

The challenge of balancing care with risk management further complicated the therapeutic relationship. P7 (Dietitian) described the pressure HCPs feel to ‘sort things out and make things right’, reflecting a solution‐focused approach, prioritising diabetes management over relational dynamics. This approach sometimes clashes with the need for empathy and understanding. P2 (Nurse) referenced the ‘policies and protocols’ that guide their actions, while P10 (Psychologist) highlighted the tension between managing physical risks and addressing patients' emotional and psychological needs: ‘[Other HCPs] hold a lot of risk, so they come from a point of view of panic’. This fear‐driven approach often resulted in HCPs feeling compelled to enforce immediate changes to address T1DE, often at the expense of understanding the underlying causes.

In their duty of care, HCPs may adopt a risk‐focused, authoritarian role rather than fostering open dialogue. P10 (Clinical Psychologist) noted this dynamic often leaves patients feeling ‘told off’ when they disclose their eating behaviours, reinforcing a sense of shame and reluctance to engage honestly with the healthcare team. This dynamic can even contribute to poorer well‐being, as P13 (Health Psychologist) explained: ‘The relationship with the diabetes team…can cause trauma’. The risk‐focused approach not only hinders the therapeutic relationship but also exacerbates patient distress, creating a cycle of mistrust and disengagement. Tensions between patients and HCPs further complicate this relationship. Patients were described as ‘difficult to work with’ (P6, Nurse), and there was an expectation of dishonesty and manipulation: ‘With eating disorders, sometimes people can be quite manipulative’ (P5, Dietitian). These attitudes, further strain the patient‐HCP relationship and impede effective screening.

While HCPs recognise the value of trust and rapport, the demands of risk management and lingering negative perceptions can create barriers to achieving this. The focus on behaviour correction, rather than understanding, risks pushing patients away, making it difficult for HCPs to effectively screen for and address T1DE.

#### 3.2.4 Theme 4: A lack of support for T1DE: ‘Where do you go?’

Whilst recognising T1DE as a widespread problem, HCPs expressed hesitation around screening due to the lack of available support, both within their teams and through referrals to specialist services. The lack of follow‐up options appeared to contribute to a sense of futility in screening for T1DE, as summarised by P4 (Dietitian): ‘I don't like just finding a problem. I would like to find a solution and support people’. Similarly, P3 (Registrar), discloses a fear screening could ‘uncover a massive problem that we don't know how to deal with’. This reluctance was rooted in the recognition many more patients likely live with T1DE than are currently acknowledged, but the absence of effective support mechanisms led to concerns identifying these cases creates additional challenges without clear solutions.

Participants overwhelmingly felt T1DE screening would be unproductive due to a lack of support options. P12 (Midwife) joked she would refer individuals to ‘anyone who will listen (laughs)’, highlighting a broader issue that both patients and HCPs feel unheard. The separation between physical and mental health services exacerbated this issue, leaving both patients and HCPs feeling abandoned. P8 (Nurse) summarises:

you have to go to another department to do that bit and another department to do that bit. And it's very frustrating. It's like, you know, you can see this lady is struggling with these, you know, mental health issues and surely you should say, get other people involved.

Many HCPs expressed a desire to refer T1DE patients to eating disorder services, but they described these as ‘non‐existent’ (P6, Nurse). Even when services were available, the strict criteria for access, such as meeting certain Body Mass Index (BMI) thresholds made it difficult to provide timely help, with P13 (Psychologist) stating patients with T1DE weren't ‘bad enough’. This contributed to a widely held belief screening would result in ‘often quite an, um, unproductive outcome’ (P3, Registrar).

Some HCPs described efforts to support patients with suspected T1DE within diabetes services, despite feeling inadequately equipped. P4 (Dietitian) summarises this process as ‘sink or swim’, demonstrating her sense patient support is solely down to her own efforts. P9 (Dietitian) outlines ‘rather than them having nothing and feeling abandoned […] we'll keep them on our caseload to, to offer a little bit of support’ reflecting the inadequacy of available services and the emotional strain on professionals who feel they must take on the responsibility. This sense of being unprepared led to hesitancy in screening, as HCPs questioned their ability to truly help: ‘It's unfair to say you'll help somebody when you don't have the skills’ (P8, Nurse).

The presence of a psychologist within the team was often hailed as the solution to the lack of support. When a psychologist was available, HCPs frequently referred patients to them, as described by P11 (Nurse): ‘It would always be: Ohh, let's refer to the psychologist’. For many, the absence of psychology support was seen as a ‘significant barrier’ (P5, Dietitian) to addressing T1DE. However, even when psychologists were involved, they did not always feel adequately prepared to address T1DE. P10 (Psychologist) stated, ‘I'm not up on all of that [disordered eating] stuff’, highlighting the challenges of supporting T1DE for all HCPs.

The absence of appropriate referral pathways, combined with a fragmented healthcare system, leads to a reluctance to screen for T1DE. While the presence of a psychologist is often seen as a potential solution, the broader system's failure to integrate mental and physical health services leaves HCPs feeling unsupported and patients underserved.

## 4 DISCUSSION

This study is the first to explore HCPs’ attitudes towards screening for T1DE within adult services. It reveals a nuanced understanding of T1DE as a complex and overwhelming issue, which instils hesitancy in HCPs when considering screening. Effective screening is seen as reliant on a strong therapeutic relationship, yet is often deemed futile due to the perceived lack of adequate support services. These challenges collectively contribute to the underutilisation of T1DE screening, underscoring the urgent need for changes in practice to better identify individuals with T1DE.

HCPs in this study struggled to balance the complexity of T1DE alongside other clinical duties. They perceived T1DE as inevitable within their services, driven by the intense focus on food in T1D management, attributing T1DE to factors beyond T1D, such as prior trauma. This externalisation may function to distance HCPs from the responsibility of screening and managing T1DE and aligns with patterns observed in other contexts, where HCPs attribute disordered eating to external factors.^18^ Moreover, the reliance on visible symptoms, such as gender or Body Mass Index (BMI), as indicators of T1DE reflects an oversimplification of the complex realities of T1DE. In T1D weight fluctuations can be misleading, and BMI is a particularly poor indicator of eating disorders,^19^ therefore reliance on surface‐level symptoms may contribute to healthcare disparities. HCPs may employ coping strategies to manage the overwhelming complexity of T1DE, which contribute to a cycle of limited screening and delayed intervention. Drawing on theories of cognitive dissonance^20^ this behaviour can be seen as an attempt to resolve the discomfort of recognising a complex problem that feels beyond their control. Addressing these cognitive biases and enhancing training on the subtle presentations of T1DE may be critical steps in improving screening practices and outcomes for patients.

Whilst not reported here, HCPs referenced the well‐documented practical challenges of working within a resource‐strained health system.^21^, ^22^ These systematic constraints impacted their ability to provide the necessary time and support for T1DE screening. Despite prior research advocating for a multidisciplinary approach in the assessment and management of T1DE,^23^, ^24^ HCPs in this study felt isolated in their responsibilities. This sense of being alone left them feeling vulnerable, fearing broaching the subject of T1DE might damage the patient‐provider relationship. HCPs also expressed concerns asking about T1DE might cause harm, akin to the anxieties often associated with discussing suicide.^25^ However, research on suicide prevention indicates asking about these sensitive issues does not cause harm; rather, it can strengthen the patient‐provider relationship and enhance trust.^26^ Addressing these fears and providing HCPs with the necessary support may mitigate the anxiety around T1DE screening. Additionally, fostering a more collaborative, multidisciplinary approach to T1DE management may help alleviate the sense of isolation HCPs experience, ensuring screening and subsequent interventions become a shared responsibility rather than a solitary burden. Such an approach is currently being piloted in eight NHS sites across England which consider both the physical and mental health needs of those with T1DE and could improve outcomes.^27^

A barrier to screening observed in this study appears to be closely linked to a fear identifying T1DE would require substantial intervention. This fear is compounded by a sense of being ill‐equipped to manage this due to significant constraints on resources. The lack of dedicated support services exacerbates this issue. In the absence of readily available referral pathways or specialist services for T1DE,^28^ HCPs often resorted to directing patients to websites, highlighting a gap in the support infrastructure. HCPs may experience a sense of professional inadequacy, as they struggle to address a complex issue without appropriate resources or guidance. This situation underscores a critical need for systemic changes to better support HCPs and patients in screening for T1DE. Developing and implementing dedicated referral pathways and specialised support services would not only enhance the capacity of HCPs to manage such cases but also provide patients with the targeted care they need.

### 4.1 Strengths, limitations and future research

The study's strengths include the participation of a diverse range of HCPs. However, the sample was primarily comprised of female HCPs. Given female healthcare professionals are more likely to have a special interest in disordered eating^29^ this may have influenced willingness to volunteer for the study. Additionally, all participants were recruited from specialist diabetes services in secondary care settings and had prior experience working with individuals with T1DE and recognised the need for change. Future research may benefit from the inclusion of HCPs in primary care settings or settings without specialist diabetes expertise, who offer care to those with T1DE. There is some indication these interactions with non‐specialist teams may exacerbate T1DE presentations, meaning further work here should be prioritised.^27^

### 4.2 Clinical implications

These discussions did not identify a single best screening tool for T1DE but helped establish a strong foundation for healthcare providers by enhancing awareness, knowledge, and communication about T1DE, ultimately supporting the implementation of screening practices. This study identifies key areas for improvement, starting with the need for enhanced training on T1DE. Participants expressed a desire for increased knowledge on topics such as the intersection of mental health and T1D. Currently, there is no evidence‐based HCP training for T1DE. Existing training programs for eating disorders have shown success in increasing healthcare professionals' willingness to treat, suggesting a similar approach could be beneficial for T1DE.^30^

In addition to knowledge‐based training, participants indicated a need for skills‐based training to build confidence in addressing T1DE with patients. HCPs reported difficulty initiating conversations about disordered eating, emphasising the importance of early intervention and overcoming fears of causing harm. Training programs could include practical guidance, example questions to facilitate discussion, and role‐play scenarios to help healthcare professionals practice in a safe environment. Clearer guidelines for managing T1DE within teams and resources for clinicians to share with patients would also help streamline the process. Embedding psychologists within diabetes teams may be helpful. A psychologist could offer staff support, participate in complex case discussions, and provide consultation to ensure a psychological perspective is integrated into patient care. Current specialist T1DE services have highlighted the benefit of sharing the expertise and experience of multidisciplinary team members, including psychologists, in this way.^26^ Such an approach requires support from the healthcare system, so teams have the necessary resources and guidelines to facilitate this.

## 5 CONCLUSION

HCPs infrequently utilise screening tools or discuss T1DE in adult services. Instead, they rely on clinical indicators and biases to identify at‐risk individuals due to a fear of patients' reactions to being asked questions about T1DE, and a perception of a lack of support. Training to increase awareness of T1DE‐specific screening tools, and skills‐based training focused on conversations about T1DE, could benefit HCPs and improve the identification of those at risk. Furthermore, there is a need for clear clinical guidelines and referral pathways to support HCPs in identifying T1DE.

## FUNDING INFORMATION

The authors declare that no funding was received for this research.

## CONFLICT OF INTEREST STATEMENT

There are no conflicts of interest to declare.

## Supporting information


Data S1.

Data S2.
