# Supplementary material for: Exploring healthcare professionals' attitudes to screening for disordered eating in type 1 diabetes
Source: Diabet Med. 2025 Feb 13;42(5):e70003. doi: 10.1111/dme.70003 (PMC12006562; doi:10.1111/dme.70003)
Supplement: Supplementary file 1 — Data S1. [file DME-42-e70003-s001.docx]

**Supplementary file: Interview guide**

**Introductory Question:** Let’s start by talking about your job, can you tell me about the service you work for and what your current role involves? How long have you been in this role? *Team make-up, psychologists within team.* What does a typical week in your work involve?

**Key Question #1:** Could you tell me about your thoughts around mental health in T1D? *What type of problems exist? How prevalent are they?*

**Key Question #2:** Can you tell me anything you know about disordered eating in T1D? *How did you gain this knowledge?*

**Key Question #3:** Thinking about your clinic appointments, have you been concerned about disordered eating in your patients? *Why were you concerned? Did the concerns come from you, or from others? (e.g. patient themselves, etc). What did you do about these concerns?*

**Key Question #4:** How able do you feel to ask patients about their relationships with food, body image or disordered eating in clinical appointments? *Is this important, how do you do this, how do you feel about doing this? Whose role is this?*

**Key Question #5:** What, if any, are the challenges in relation to asking about disordered eating in practice? *Does the service you work for provide guidance/support on this?*

**Key Question #6:** Are you aware of any measures which screen for disordered eating in diabetes? *Do you use any? Reasons for not using?*
