# Supplementary material for: Exploring healthcare professionals' attitudes to screening for disordered eating in type 1 diabetes
Source: Diabet Med. 2025 Feb 13;42(5):e70003. doi: 10.1111/dme.70003 (PMC12006562; doi:10.1111/dme.70003)
Supplement: Supplementary file 2 — Data S2. [file DME-42-e70003-s002.docx]

**Supporting information: COREQ quality guidelines not for publication**

| **Domain** | **Item** | **Description** | **Manuscript Text** |
| --- | --- | --- | --- |
| **1. Research Team and Reflexivity** | 1.1 **Personal Characteristics** | Describe the research team members’ qualifications, experience, and any relevant personal characteristics. | Team members qualifications are detailed on the title page (PhD, PsychD, MBBS).  *“In this study, all researchers are female, with professional backgrounds in T1D healthcare and/or psychology. One has lived experience of T1D (RS) and another (KF) has personal experience of a bereavement due to diabetes.”* |
|  | 1.2 **Relationship with Participants** | Describe the relationship between researchers and participants, including any biases or influences. | *Interviews were conducted by a trainee clinical psychologist (KF) who had no prior relationship with participants. KF’s psychologist role may have influenced the way participants discussed mental health topics.* |
|  | 1.3 **Researcher's Role** | Explain the role of the researcher in the research process (e.g., data collection, analysis). | *Interviews were conducted by a trainee clinical psychologist (KF) who had no prior relationship with participants. KF’s psychologist role may have influenced the way participants discussed mental health topics. Despite this, participants displayed a notable level of comfort and openness, suggesting that the interviewer's professional background helped foster trust and facilitated rich data collection. The team engaged in reflexive practices to critically examine how their experiences and positions influenced their analysis, ensuring that both individual and broader contextual factors were considered.* |
| **2. Study Design** | 2.1 **Qualitative Methodology** | Specify the qualitative method(s) used (e.g., interviews, focus groups). | *The 60-minute interview covered the clinicians' understanding of mental health and T1D, their experiences with T1DE, use of screening tools in clinical practice, and their views on discussing T1DE with patients.* |
|  | 2.2 **Research Purpose** | State the purpose of the research and the specific research questions being addressed. | *Adults with T1DE face distinct challenges, such as increased autonomy over diabetes care and unique psychosocial stressors, which may exacerbate disordered eating behaviours. This study addresses the gap by exploring HCPs perspectives and attitudes toward T1DE screening in adult T1D services.* |
| **3. Sampling** | 3.1 **Sampling Strategy** | Describe the sampling strategy used to select participants (e.g., purposive, convenience). | *Purposive sampling ensured diversity across HCP roles.* |
|  | 3.2 **Sample Size** | Provide the rationale for the chosen sample size, including the final number of participants. | *Critical realism emphasises understanding the underlying mechanisms and structures that shape human behaviour. By conducting interviews with 13 healthcare professionals, we obtained rich, detailed narratives that provided insight into their beliefs and experiences regarding disordered eating screening in adults with Type 1 diabetes.* |
| **4. Data Collection** | 4.1 **Data Collection Method** | Describe the methods used for data collection (e.g., interviews, focus groups). | *A semi-structured interview guide (supplementary file 1) was created with input from two non-participating T1D healthcare professionals and pilot-tested with three participants, whose data were included in the analysis as no changes were needed. The 60-minute interview covered the clinicians' understanding of mental health and T1D, their experiences with T1DE, use of screening tools in clinical practice, and their views on discussing T1DE with patients.* |
|  | 4.2 **Data Collection Instruments** | Describe any instruments used (e.g., interview guides, questionnaires). |  |
|  | 4.3 **Pilot Testing** | Indicate whether pilot testing was conducted for the data collection methods. |  |
| **5. Data Analysis** | 5.1 **Data Analysis Process** | Describe the process of data analysis (e.g., thematic analysis, coding). | *Audio recordings were transcribed, capturing communication nuances such as pauses or laughter. Identifiable information, such as names or locations, were redacted to protect anonymity. Transcripts were uploaded to NVivo to manage data analysis.*  Reflexive thematic analysis16 began with data familiarisation, involving re-reading transcripts and re-watching video recordings to capture verbal and non-verbal cues. This allowed for a deeper understanding of the context in which the data were generated. Transcripts were then open-coded, considering both semantic meaning, and latent, underlying meaning, interpreted by the research team. Theme development involved discussion within the research team to identify, interpret and refine underlying patterns within the data. |
|  | 5.2 **Software Used** | Specify any software used for data analysis (e.g., NVivo, Atlas.ti). |  |
| **6. Findings** | 6.1 **Findings and Interpretation** | Present key findings and interpretations that emerge from the data analysis. | Examples can be found throughout the results section of the manuscript, illustrative examples are presented here:  *Four themes were identified: (1) “A bit of a black hole”: Understanding T1DE, (2) “My fear is...am I overstepping the line”: Asking about T1DE, (3) “A backward step”: Patient -provider relationships and (4) “Where do you go?”: Lack of support for T1DE.*  *When HCPs suspect T1DE, they often relied on external evidence to validate their concerns prior to initiating a discussion with the patient. This approach was seen as means to bolster HCP confidence and provide a rationale for addressing the issue. One participant described the process of gathering evidence as being like a detective: “Checking back with the pharmacy…So you’ve got some evidence to support your suspicions” (P7, Dietitian). Another described this process as “a solid piece of evidence almost to back up why you’re asking that question and then kind of makes you feel more confident” (P11, Nurse). While this approach could provide a sense of security, it also suggests that HCPs may need more confidence and support in trusting their clinical judgment.* |
|  | 6.2 **Thick Description** | Provide rich, detailed descriptions of the findings to enhance understanding of the context. |  |
| **7. Discussion** | 7.1 **Comparison with Existing Literature** | Discuss how the findings compare with existing literature and implications for practice. | Examples can be found throughout the discussion section of the manuscript, illustrative examples are presented here:  *This externalisation may function to distance HCPs from the responsibility of screening and managing T1DE and aligns with patterns observed in other contexts, where HCPs attribute disordered eating to external factors.17 Moreover, the reliance on visible symptoms, such as gender or Body Mass Index (BMI), as indicators of T1DE reflects an oversimplification of the complex realities of T1DE. In T1D weight fluctuations can be misleading, and BMI is a particularly poor indicator of eating disorders,18 therefore reliance on surface-level symptoms may contribute to healthcare disparities.*  *Additionally, all participants were recruited from specialist diabetes services in secondary care settings, and had prior experience working with individuals with T1DE and recognised the need for change. Future research may benefit from inclusion of HCPs in primary care settings or settings without diabetes expertise, who offer care to those with T1DE. There is some indication that these interactions with non-specialist teams may exacerbate T1DE presentations, meaning further work here should be prioritised.26*  *Training programs could include practical guidance, example questions to facilitate discussion, and role-play scenarios to help healthcare professionals practice in a safe environment. Clearer guidelines for managing T1DE within teams and resources for clinicians to share with patients would also help streamline the process.* |
|  | 7.2 **Limitations** | Acknowledge any limitations of the study, including potential biases. |  |
|  | 7.3 **Implications for Practice** | Discuss the practical implications of the findings for healthcare, policy, or future research. |  |
